# *CsRAB*, a R2R3-MYB transcription factor from purple tea (*Camellia sinensis*), positively regulates anthocyanin biosynthesis

**DOI:** 10.3389/fpls.2024.1514631

**Published:** 2024-12-06

**Authors:** Hualing Wu, Yayan Pan, Erdong Ni, Dandan Qin, Kaixing Fang, Qing Wang, Chengwei Yang, Ming Luo, Jun Liu

**Affiliations:** ^1^ Tea Research Institute, Guangdong Academy of Agricultural Sciences, Guangdong Provincial Key Laboratory of Tea Plant Resources Innovation and Utilization, Guangzhou, Guangdong, China; ^2^ School of Life Science, South China Normal University, Guangzhou, Guangdong, China; ^3^ Guangdong Provincial Key Laboratory of Applied Botany & Agriculture and Biotechnology Research Center, South China Botanical Garden, Chinese Academy of Sciences, Guangzhou, China

**Keywords:** secondary metabolism, anthocyanins, R2R3-MYB gene, tea (*Camellia sinensis*), purple tea

## Abstract

In tea (*Camellia sinensis*), anthocyanins are important secondary metabolites that are linked to leaf color. Anthocyanin biosynthesis is a complex biological process, in which multiple genes including structural and regulatory genes are involved. Here, we describe the cloning and characterizing of a new R2R3-MYB transcription factor gene, *CsRAB*, isolated from purple tea variety ‘*Hongfei*’. Consistent with its predicted role as a transcription factor, the CsRAB protein localized to nuclei when expressed in onion (*Allium cepa*) epidermal cell. A dual-luciferase reporter assay demonstrated that CsRAB acts as a transcriptional activator *in vivo*. *CsRAB* overexpression in *Arabidopsis* seedlings led to higher expression levels of anthocyanin biosynthesis-related genes, and consequently, purple stems and higher anthocyanin contents were exhibited in overexpressing lines compared to wild type. The results indicated that *CsRAB* plays critical roles in positively regulating anthocyanins biosynthesis in tea plants.

## Introduction

Tea (*Camellia sinensis*) is a dicotyledonous diploid plant belonging to the *Theaceae* family. It is one of the most important commercial crops owing to its high contents of secondary metabolites, such as flavonoids, theanine, and caffeine. The flavonoids represent the largest group, including flavones, flavonol, anthocyanins, catechins, and procyanidines (Owuor and Obanda, 2007; Winkel-Shirley, 2001). Purple tender shoots are a feature of tea plants. They are occasionally seen when ordinary tea varieties are experiencing external environmental stress or seasonal changes. In some special purple tea varieties, however, a stable and heritable purple color is seen in the topmost buds and leaves (including buds and approximate three to four adjacent young leaves) of tender shoots throughout the year, owing to the abundant levels of anthocyanins synthesized and (1) accumulated in these organs (Joshi et al., 2017; Lai et al., 2016). As more health-promoting functions of anthocyanins in purple tea have been elucidated, breeding and product development efforts for these distinctive kind of tea plants have increased (Krga and Milenkovic, 2019; Li et al., 2016; Tan et al., 2020). Anthocyanins form a group of the most important metabolites in the flavonoid biosynthetic pathway (Zhao and Tao, 2015). As water-soluble pigments, anthocyanins exist extensively in flowers, stems, leaves and fruits of plants. The anthocyanin metabolic pathway in plants depends on the expression levels of two classes of genes. The first group contains structural genes, which encode catalyzing enzymes involved in anthocyanin synthesis, such as *phenylalanine ammonia lyase* (*PAL*), *cinnamic acid 4-hydroxylase* (*C4H*), *4-coumaroyl CoA ligase* (*4CL*), *chalcone synthase* (*CHS*), *chalcone isomerase* (*CHI*), *flavanone 3-hydroxylase* (*F3H*), *flavonoid-3’-hydroxylase* (*F3’H*), *flavonoid-3’,5’-hydroxylase*, *dihydroflavonol 4-reductase* (*DFR*), *anthocyanin synthase* (*ANS*) and *UDP-glucose: flavonoid 3-O-glucosyltransferase* (*UFGT*) (Holton and Cornish, 1995; Tanaka and Ohmiya, 2008). The second group contains regulatory genes, mainly including *MYB*, *basic helix loop helix* (*bHLH*) and *WD40-repeat proterin*, such as *AtTT2* (*R2R3-MYB*), *AtTT8* (*bHLH*), and *AtTTG1* (*WD40-repeat protein*) in *Arabidopsis thaliana*, which encode transcription factors and regulate temporal and spatial expression patterns of structural genes in response to external stimuli (Baudry et al., 2004).

As members of a large transcription factors family, MYB proteins participate in the regulation of many plants developmental and defense response pathways (Xie et al., 2006). Plant MYB proteins are classified into three subfamilies on the basis of their N-terminal MYB domains structures, which contain one to three adjacent repeats (R1, R2, and R3), R2R3-MYB and R1R2R3-MYB, as well as a heterogeneous group of MYB-related proteins that have one repeat or none (Dubos et al., 2010). Previous MYBs studies demonstrated that R2R3-MYBs may regulate the synthesis of many secondary metabolites (Chen et al., 2006; Vom Endt et al., 2002). In particular, R2R3-MYBs play critical roles in specifically recognizing and regulating structural genes, resulting in the promotion or repression of anthocyanin synthesis in many species, such as maize (*Zea mays*) (Paz-Ares et al., 1987), petunia (*Petunia integrifolia*) (Albert et al., 2011), *Arabidopsis* (*Arabidopsis thaliana*) (Borevitz et al., 2000; Nguyen et al., 2015), apple (*Malus × domestica*) (Espley et al., 2007), grapes (*Vitis vinifera*) (Walker et al., 2010), pears (*Pyrus pyrifolia*) (Feng et al., 2010) and *Chrysanthemum* (*Chrysanthemum morifolium*) (Liu et al., 2015).

Efforts to identify the genes involved in the anthocyanin biosynthetic regulation in tea plants have been made in recent years (Chen et al., 2020; Shi et al., 2021; Sun et al., 2016; Zhu et al., 2021). More recently, by analyzing the transcriptome of purple tea cultivars ‘*Zijuan*’ and ‘*Ziyan*’, a group of key structural genes potentially involved in the anthocyanin biosynthetic and glycolytic pathways has been uncovered (Chen et al., 2015; He et al., 2021; Li et al., 2016; Mei et al., 2020; Sun et al., 2016; Wu et al., 2012; Xu et al., 2014). To date, many *MYB* genes involved in anthocyanin biosynthesis and accumulation in tea have also been reported, such as *CsAN1*, *CsMYB1*, *CsMYB2*, *CsMYB4a*, *CsMYB5a*, *CsMYB5b*, *CsMYB5e*, *CsMYB6A*, *CsMYB75, CsMYB90*, *CsMYB26*, *CsMYBL2a* and *CsMYBL2b* (Cai et al., 2022; He et al., 2018; Jiang et al., 2018; Li et al., 2017; Sun et al., 2016; Wang et al., 2018, 2019, 2024; Wei et al., 2019; Zhao et al., 2023; Zheng et al., 2019). However, the underlying molecular regulation mechanisms of the anthocyanin synthesis pathway varies based on varieties. In the present study, the phenotype, and chemical characteristics of ‘*Hongfei*’ were analyzed, indicating ‘*Hongfei*’ is a purple tea variety with stable and heritable high anthocyanin accumulation and different from ‘*Zijuan*’. Significant higher expression levels of a new *MYB* gene, *CsRAB*, and five key anthocyanin structural genes of the purple leaves in ‘*Hongfei*’ suggesting that they play important roles in anthocyanin synthesis and accumulation in tea plants. To decipher the molecular mechanisms underlying the high anthocyanin level in tender shoots of purple tea variety ‘*Hongfei*’, we isolated the full-length cDNA and genomic sequences of a *R2R3-MYB* gene, named *CsRAB*, which is highly expressed in purple leaves. A phylogenetic analysis demonstrated that CsRAB is distantly related to other previously reported MYB proteins in tea plants. CsRAB localized to cell nuclei and acted as a transcriptional activator *in vivo*. When overexpressed in the model plant *Arabidopisis thaliana*, *CsRAB* markedly upregulated the expression of key structural genes involved in the anthocyanin biosynthesis and consequently increased anthocyanin contents. Our results reveal a novel regulatory pathway of anthocyanin biosynthesis in tea plants, and they provide more insights into the regulation of anthocyanin synthesis-related metabolism.

## Materials and methods

### Plant materials

**Figure 1:**
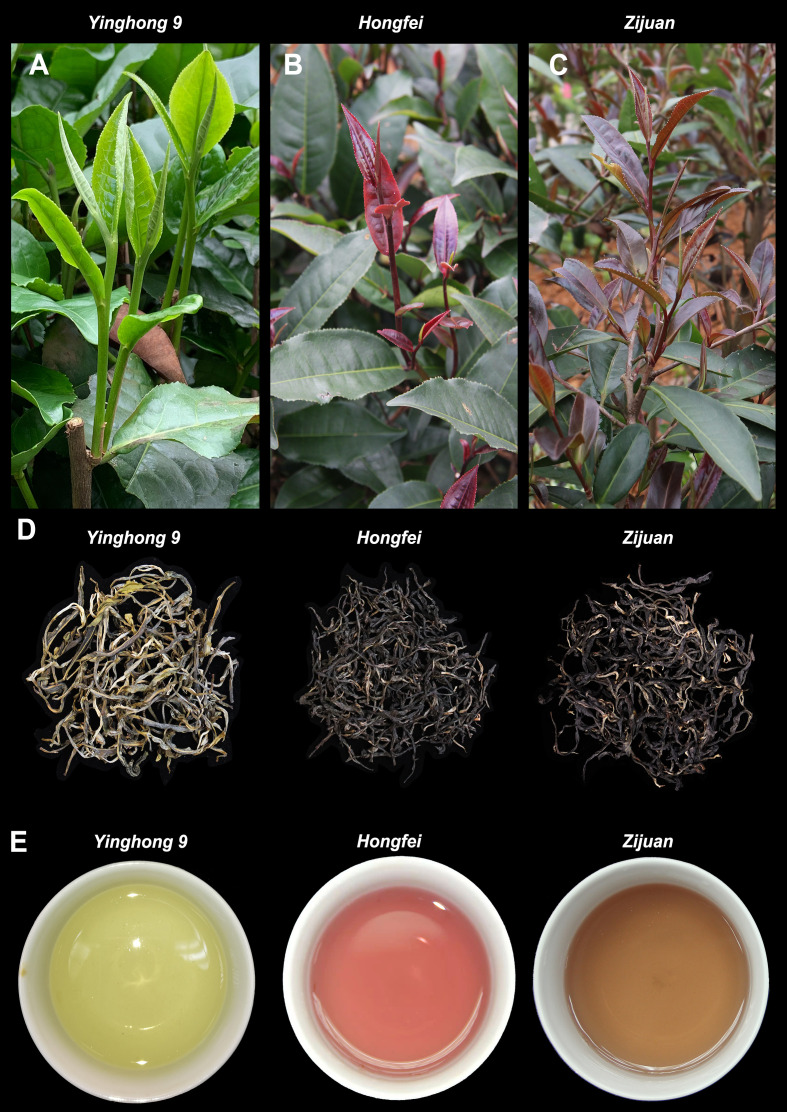
Comparison of phenotype and tea soup of *Camellia sinensis* varieties ‘*Yinghong 9*’, ‘*Hongfei*’ and ‘*Zijuan*’. **(A)** Young shoots and leaves of ‘*Yinghong 9*’. **(B)** Young shoots and leaves of ‘*Hongfei*’. **(C)** Young shoots and leaves of ‘*Zijuan*’. **(D)** Green tea of ‘*Yinghong 9*’, ‘*Hongfei*’ and ‘*Zijuan*’. **(E)** Green tea soup of ‘*Yinghong 9*’, ‘*Hongfei*’ and ‘*Zijuan*’.

The tea plants ‘*Yinghong 9*’, ‘*Hongfei*’ and ‘*Zijuan*’ used in this study were grown at the experimental station of the Tea Research Institute, Guangdong Academy of Agricultural Sciences, Yingde, China. Samples used for total anthocyanins analysis were collected from the young leaves of ‘*Yinghong 9*’, ‘*Hongfei*’ and ‘*Zijuan*’ at spring, summer and autumn in Yingde, respectively. Samples used for anthocyanins profile analysis were collected from the young purple leaves of ‘*Hongfei*’ at spring in Yingde. Samples used for chemical composition analysis were collected from the young leaves of ‘*Yinghong 9*’, ‘*Hongfei*’ and ‘*Zijuan*’ at spring in Yingde, respectively. Samples used for transcriptome were collected from young purple leaves and mature green leaves of the purple tea varieties ‘*Hongfei*’ (**Figure 1**) at spring in Yingde. The sampled tissues were immediately frozen in liquid nitrogen and stored at −80°C until use.

### Extraction and quantification of anthocyanins

Total anthocyanins of plant tissue were extracted and quantified following the methods of Mehrtens et al. (2005) and Mano et al. (2007) with minor modifications. Briefly, 1 mL of 1% [v/v] HCl in methanol was added to 0.3 g of fresh plant tissue. Samples were incubated for 18 h at 22°C under moderate shaking (100 ×g). After centrifugation (12,000×g at room temperature for 3 min), 0.4 mL of the supernatant was added to 0.6 mL of acidic methanol. The absorption levels of the extracts at wavelengths of 530 and 657 nm were detected photometrically (Beckman DU 640 Spectrophotometer, USA). The anthocyanin content was calculated using the following formula: Q_Anthocyanins_ = (A_530_−0.25A_657_) × M**^−^** ^1^, where Q_Anthocyanins_ represents the anthocyanin amount, A_530_ and A_657_ represent the absorption values at the indicated wavelengths, and M represents the fresh weight (g) of the seedlings used for extraction. The obtained data were the means of three biological independent replicates.

### Anthocyanins profile analysis using LC-MS

Anthocyanins profile analyzed following the methods of Cooney et al. (2010) with minor modifications. Briefly, 800 μL methanol was added to 50 mg of fresh plant tissue and 10 μL internal standard (dichlorophenylalanine, 2.5 mg/mL), and then placed in the tissue grinding machine at 65 Hz for 90 s. Samples were vortexed 30 s, and centrifuged at 12, 000 RPM for 15 min at 4°C. 200 μL supernatant was transferred to vial for Liquid Chromotography with Mass Spectrometry (LC-MS) analysis (ThermoQuest, Finnigan, San Jose, CA, USA). The temperature of the column oven was maintained at 40°C. The solvent A was water (0.1% formic acid, v/v) and solvent B was methanol (0.1% formic acid, v/v). Flow rate was 0.3 mL/min, and the injection volume was 6 µL.

### Chemical composition analysis

Total tea water extraction, total tea polyphenols, and free amino acids were extracted and quantified by the Chinese National Standard GB/T 8305-2002, GB/T 8313-2008, GB/T 8314-2002, respectively (Wu et al., 2012). The determination of caffeine and catechin contents was performed by High Performance Liquid Chromatography (HPLC) (Wu et al., 2012) with minor modifications. 0.2 g tea powder was extracted with 70% methanol twice, then filtered through a 0.22 μm microporous filter. The caffeine and catechins were determined by high HPLC (Agilent, HPLC 1200, USA).

### Isolation of the full-length cDNA and genomic DNA of *CsRAB*

Total RNAs were extracted from fresh purple leaves of ‘*Hongfei*’ using a TRIzol kit (Invitrogen, USA) and then treated with DNase I (Fermentas, USA) for removing genomic DNA. The integrity and quality of RNAs were confirmed by electrophoresis in 1% agarose gels. The full-length *CsRAB* cDNA was isolated using the RACE method. Based on the sequence of the *CsRAB* EST obtained in our previous work, four specific primers, 3′GSP1 and 5′GSP1 and nested primers 3′GSP2 and 5′GSP2 (**Supplementary Table S1**), were designed using Premier 5.0 (http://www.premierbiosoft.com) and synthesized at the Beijing Genomics Institute (Shengzhen, China) to amplify the 3′ and 5′ ends of *CsRAB.* For 3′RACE, first-strand cDNA was synthesized using M-MLV reverse transcriptase (Promega, USA) in according with the manufacturer’s instructions and primer APT containing the sequence the adapter primer AP (**Supplementary Table S1**). Then, the forward primer 3′GSP1 and reverse adapter primer AP were used for the first round of PCR amplification using the synthesized first-strand cDNA as the template, followed by a second round of nested PCR using the PCR product from the first round as template and primers 3′GSP2 and AP. The amplification of 3′ end sequence was performed under the following conditions: 94°C for 3 min, followed by 30 cycles of amplification at 94°C for 30 s, 52°C for 30 s, and 72°C for 90 s; followed by an extension for 10 min at 72°C. The 5′RACE was performed in accordance with the instructions of the SMART™ RACE amplification kit (Clontech, USA). The PCR products were then purified, cloned into the pMD18-T vector (TaKaRa, China), sequenced and validated. Subsequently, the *CsRAB* full-length cDNA sequence was obtained by aligning and assembling the sequences of 3′- and 5′- sequences using DNAMAN software version 6.0, verified through PCR amplification using the end primer pair CDF and CDR (**Supplementary Table S1**) and further sequencing.

Genomic DNA was also isolated from the purple leaves of ‘*Hongfei*’ following a modified CTAB method described by Dellaporta et al. (1983). Two gene-specific primers, denoted GDF and GDR (**Supplementary Table S1**), designed on the basis of cDNA sequence, were used to amplify the *CsRAB* genomic sequence.

### Bioinformatics analysis

The protein molecular weight prediction and isoelectric point were calculated using ExPASY (http://web.expasy.org/protparam/). A motif search was performed online using the NCBI protein analysis program (http://blast.ncbi.nlm.nih.gov/Blast.cgi). A multiple amino-acid sequence alignment and phylogenetic tree (by NJT with 500 bootstrap replicates) of the predicted products of the MYB genes were performed using Clustalx1.83 and, constructed using MEGA software version 7.0. The alignment image was generated using GeneDoc software.

### Sub-cellular localization of CsRAB

The *CsRAB* gene specific primers, pGFPF and pGFPR (**Supplementary Table S1**), which incorporated a *Kpn* I and a *Bam*H I restriction site, respectively, were designed to amplify the *CsRAB* gene using the first-strand cDNA applied in 3′RACE as the template. PCR amplification carried out as follows: 94°C for 3 min, followed by 30 cycles of amplification at 94°C for 30 s, 60°C for 30 s, and 72°C for 90 s; followed by an extension for 10 min at 72°C. The products were digested and ligated into the pBEGFP vector, containing *EGFP* fragment, to generate the p35S*::CsRAB*-*EGFP* recombinant plasmid. After confirmation by restriction enzyme digestion and sequencing analysis, the recombinant plasmid and the pBEGFP vector were introduced individually into onion epidermal cells by gene gun bombardment, incubated on MS medium for 12 h at 22°C in darkness, and visualized under a fluorescence microscope (Zeiss LSM 710 meta, Germany). All the transient expression assays were repeated three times. The observed results were pictured and displayed using LSM 5 Image Browse software.

### Dual-luciferase reporter assay

A dual-luciferase reporter analysis was performed according to the procedures of Han et al. (2016). The dual REN**/**LUC reporter and effectors were co-transformed into protoplast of *Arabidopsis* by PEG-mediated transformation. LUC and REN luciferase activities were analyzed, and the transcription activation capability of CsRAB was indicated by the ratio of LUC to REN.

### Generation of Arabidopsis transgenic lines overexpressing *CsRAB*

The construct for overexpressing *CsRAB* was created using Gateway^®^ technology through the two-step recombination reactions, BP and LR, following the instructions of the Gateway^®^BP Clonase^™^ II Enzyme Mix and Gateway^®^-LR Clonase^™^ II Enzyme Mix (Invitrogen, USA). Using the cDNA sequence of *CsRAB* gene, we designed four specific primers, BPF and BPR, which were used in the BP reaction, and LPF and LPR, which were used in the LP reaction (**Supplementary Table S1**). For the BP reaction, the attB recombination sites were integrated into the two ends of the *CsRAB* ORF by PCR amplification, and then, they were recombined in an intermediate vector pDONOR221 containing two attP sites to form a recombinant vector containing two attL sites. Subsequently, for the LP reaction, the recombinant vector obtained in the BP reaction was replaced by the pB2WG7 vector with two attR sites to construct the plant overexpression vector pB2WG7-CsRAB, which was then introduced into *A*. *tumefaciens* EHA105. All the combinatory vectors were confirmed by sequencing.

Using the floral dip method (Clough and Bent, 1998), *A. tumefaciens* harboring the pB2WG7-CsRAB vector was transformed into *Arabidopsis* (Col-0). Transgenic seedlings were selected on MS media containing 0.001% Basta. They were then transferred into soil and grown at 22°C, at 70% relative humidity, under a 16-h light/8-h dark photoperiod to maturity. *CsRAB* expressing in homozygous T3 progeny plants were identified by PCR analysis.

### Gene expression analysis using qRT-PCR

RNA was isolated from leaves of tea varieties ‘*Hongfei*’ and ‘*Yinghong 9*’, from 6-d-old seedlings of three independent transgenic lines and from WT *Arabidopsis*. RNA from each sample was treated with DNaseI and reverse transcribed using 50 pmol of poly(T)_15_ primer and a M-MLV reverse transcriptase (Promega, USA). The quantitative real-time PCR (qRT-PCR) was performed using the LightCycler^®^480II Real-Time PCR System with SYBR Premix Ex Taq™ II Kit (TaKaRa, China) in accordance with the manufacturer’s protocol. Reactions were performed in triplicate using 2 μl of Master Mix, 0.5 M of each primer, 2 μl of diluted cDNA, and nuclease-free water to a final volume of 20 μl. PCR primers are listed in **Supplementary Table S1**. The qRT-PCR conditions were as follows: 95°C for 2 min, followed by 40 cycles of amplification at 95°C for 10 s, 55°C for 30 s and 72°C for 30 s, with a final extension at 72°C for 3 min. The expression levels of the tested genes were normalized using the comparative Ct method with an internal control gene (*CsActin* in tea plant and *AtActin* in *Arabidopsis*). All qRT-PCR analysis for each sample were repeated three times.

### RNA sequencing in *Arabidopsis*

Total RNA was isolated from 6-d-old transgenic and wild *Arabidopsis* seedlings grown under long-Day conditions (16-h light/8-h dark). Three independent biological samples were used to perform an RNA-seq analysis and data were analyzed, which was performed according to the procedures of Zhu et al. (2018). The cDNA libraries were constructed and sequenced using the BGISEQ-500 platform. All the full-length transcripts were annotated into three public databases, the *Arabidopsis* information resource, GO and KEGG.

## Results

### Morphological and chemical characteristic of purple tea variety ‘*Hongfei*’

**Figure 2:**
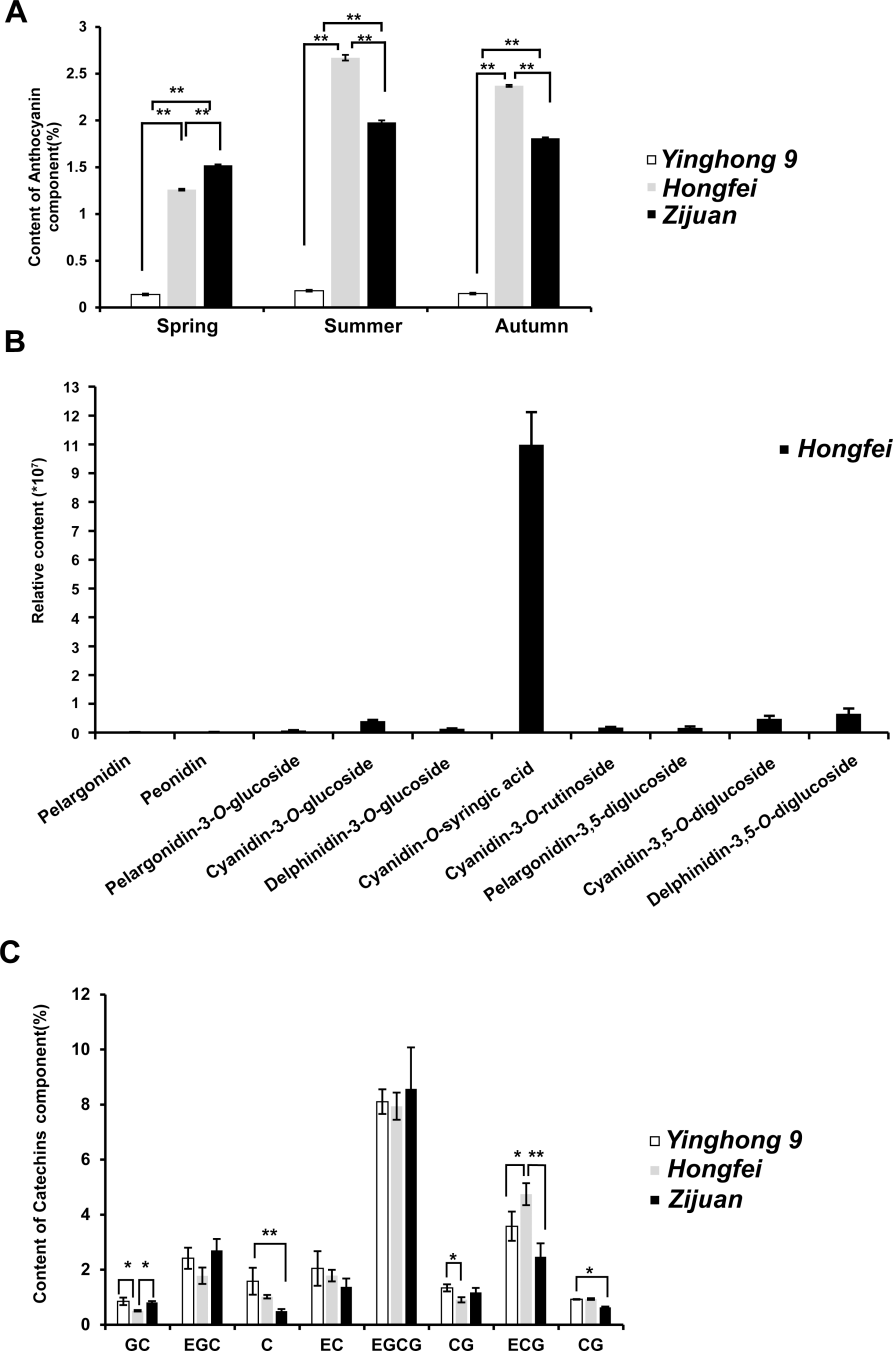
Anthocyanins and catechins characteristic of *Camellia sinensis* varieties ‘*Yinghong 9*’, ‘*Hongfei*’ and ‘*Zijuan*’. **(A)** Anthocyanins contents in steamed fresh leaves of ‘*Yinghong 9*’, ‘*Hongfei*’ and ‘*Zijuan*’ during different seasons. **(B)** Major anthocyanins of ‘*Hongfei*’. **(C)** Catechins contents in steamed fresh leaves of ‘*Yinghong 9*’, ‘*Hongfei*’ and ‘*Zijuan*’ during different seasons. Data presented are mean values of three biological repetitions. The asterisk indicates a statistically significant difference as assessed by Student’s t-test (^∗^P <0.05 and ^∗∗^P < 0.01).

‘*Hongfei*’ exhibits a stable and heritable red purple color in the topmost buds and approximate three to four adjacent young leaves of tender shoots throughout the year, which is different from purple tea cultivar ‘*Zijuan*’ with dark purple tender shoots and normal green variety ‘*Yinghong 9*’ with excellent quality (**Figures 1A–C**). All of them were selected from the Yunnan big-leaf tea group, which have a relatively similar genetic background. Differ from ‘*Yinghong 9*’, the green tea of purple varieties ‘*Hongfei*’ and ‘*Zijuan*’ exhibited shiny black (**Figure 1D**). The tea soup of ‘*Hongfei*’ displayed red purple compared with red brown of ‘*Zijuan*’ and yellow green of ‘*Yinghong 9*’ (**Figure 1E**). Anthocyanin analysis revealed ‘*Hongfei*’ displayed significantly higher anthocyanin contents than ‘*Yinghong 9*’ in three seasons (**Figure 2A**). Comparison between purple varieties ‘*Hongfei*’ and ‘*Zijuan*’ showed that the obvious higher anthocyanin contents in ‘*Hongfei*’ in summer and autumn, whereas the higher contents were detected in ‘*Zijuan*’ in spring (**Figure 2A**).The average absolute anthocyanin contents of ‘*Hongfei*’ in three seasons reach up to 2.1% of the leaf dry weight in a purple bud with two leaves, which is 13.4 times higher than that in the normal green variety ‘*Yinghong 9*’, also higher than the purple tea variety ‘*Zijuan*’ (1.77%) as well. Anthocyanin profile of ‘*Hongfei*’ was analyzed though LC-MS and the major compounds were Cyanidin-*O*-syringic acid, Delphinidin-3,5-*O*-diglucoside, Cyanidin-3,5-*O*-diglucoside, Cyanidin-3-*O*-glucoside, Cyanidin-3-*O*-rutinoside, Delphinidin-3-*O*-glucoside, Pelargonidin-3,5-diglucoside, Pelargonidin-3-*O*-glucoside, Pelargonidin, Peonidin, and among these Cyanidin-*O*-syringic acid was the dominant anthocyanin (**Figure 2B**). Chemical composition analysis of tea in steamed fresh leaves showed that average tea polyphenols content in three seasons was significantly higher in ‘*Hongfei*’ up to 37.11% compared with those of ‘*Yinghong 9*’ and ‘*Zijuan*’ (**Table 1**). However, the average amino acids and caffeine contents among three seasons were lower in ‘*Hongfei*’ compared with ‘*Yinghong 9*’and ‘*Zijuan*’ (**Table 1**). There was no significant difference among the average contents of water extract in the three varieties (**Table 1**). According to the analysis of catechin components from steamed fresh leaves through HPLC analysis, the content of ester catechin epigallocatechin gallate (EGCG) and epicatechin gallate (ECG) were most abundance in three varieties. ‘*Hongfei*’ contained less gallocatechin (GC) and much more ECG than ‘*Zijuan*’ and ‘*Yinghong 9*’ (**Figure 2C**).

**Table 1:** Major biochemical component of tea in steamed fresh leaves.

Cultivar	Season	Tea polyphenols(%)	Amino acids(%)	Caffeine(%)	Tea extract(%)
*Hongfei*	Spring	34.15 ± 0.64^d^	2.76 ± 0.09^b^	2.45 ± 0.17^c^	43.83 ± 1.29^b^
	Summer	37.66 ± 0.2^b^	1.45 ± 0.08^e^	2.3 ± 0.06^c^	44.52 ± 0.84^b^
	Autumn	39.51 ± 0.13^a^	2.18 ± 0.11^d^	2.55 ± 0.25^c^	45.9 ± 1.75^b^
	Average	37.11^c^	2.13^d^	2.43^c^	44.75^b^
*Yinghong 9*	Spring	30.15 ± 0.72^e^	3.37 ± 0.03^a^	3.28 ± 0.22^b^	41.98 ± 1.93^bc^
	Summer	31.92 ± 0.5^e^	2.55 ± 0.02^c^	2.54 ± 0.25^c^	49.88 ± 1.41^a^
	Autumn	30.67 ± 0.45^e^	2.44 ± 0.05^c^	3.19 ± 0.01^b^	42.75 ± 0.74^bc^
	Average	30.91^e^	2.79^b^	3.00^b^	44.87^b^
*Zijuan*	Spring	31.46 ± 0.14^e^	3.36 ± 0.06^a^	2.8 ± 0.46^b^	46.1 ± 1.51^b^
	Summer	30.51 ± 1.26^e^	2.79 ± 0.07^b^	2.31 ± 0.13^c^	45.79 ± 1.26^b^
	Autumn	29.31 ± 0.28^f^	2.15 ± 0.23^d^	3.93 ± 0.06^a^	43.67 ± 0.78^b^
	Average	30.43^e^	2.77^b^	3.01^b^	45.19^b^

Data are means ± SD of three replicates. ^a,b,c,d,e,f^Different letters in the same column indicate significant differences between mean values (*p* < 0.05).

### Cloning and sequence analysis of *CsRAB*

**Figure 3:**
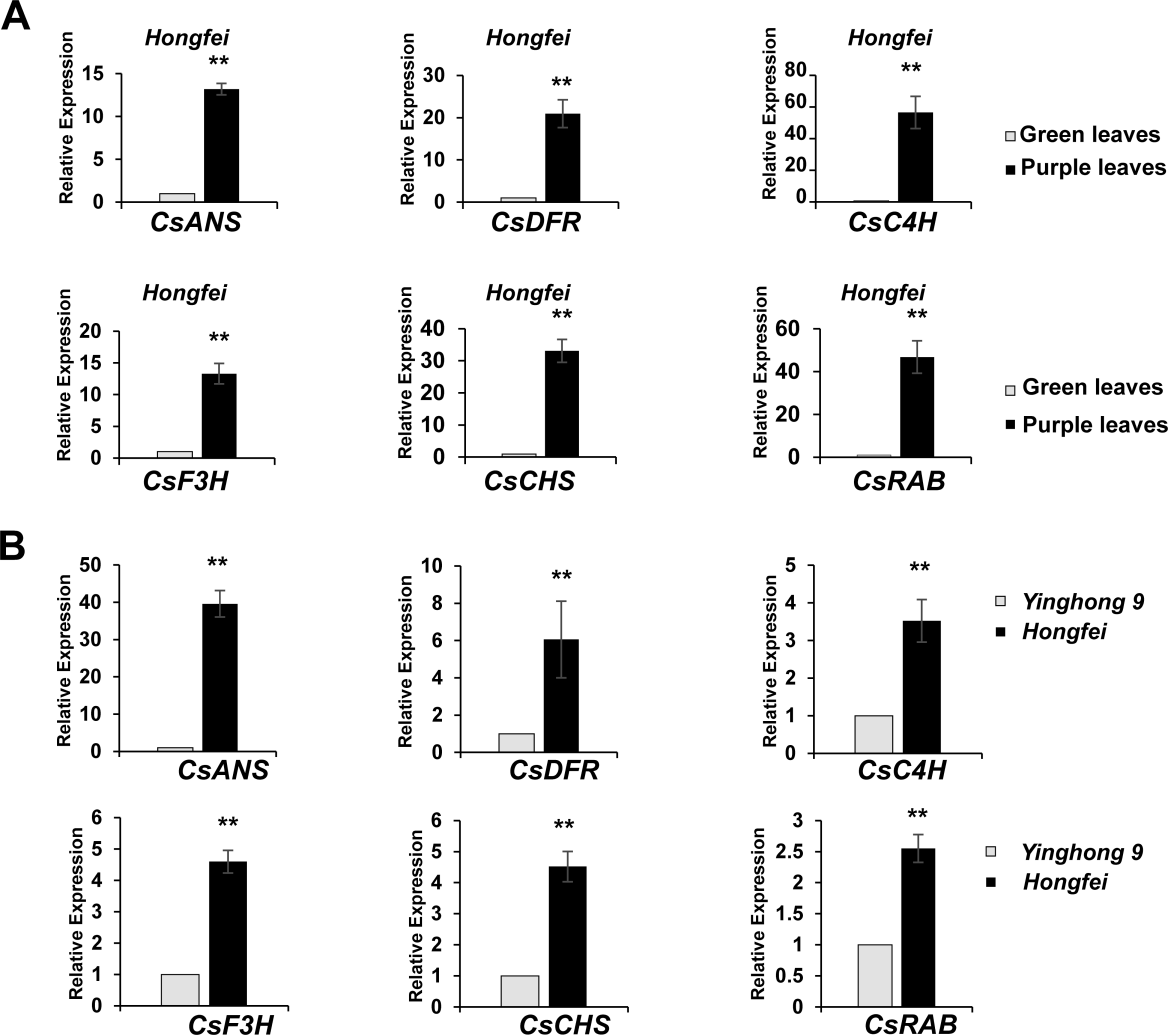
Expression of the genes encoding the anthocyanin biosynthetic enzymes and *CsRAB*. **(A)** The quantitative real-time PCR (qRT-PCR) of anthocyanin biosynthesis related genes in the purple and green leaves of the purple tea varieties ‘*Hongfei*’. **(B)** The qRT-PCR of anthocyanin biosynthesis related genes in the purple tea varieties ‘*Hongfei*’ and normal green variety ‘*Yinghong 9*’. All qRT-PCRs were normalized using the Ct value of the *CsActin* gene. Error bars indicate standard deviation calculated from three biological replicates. The asterisk indicates a statistically significant difference as assessed by Student’s t-test (^∗∗^ *P* < 0. 01).

**Figure 4:**
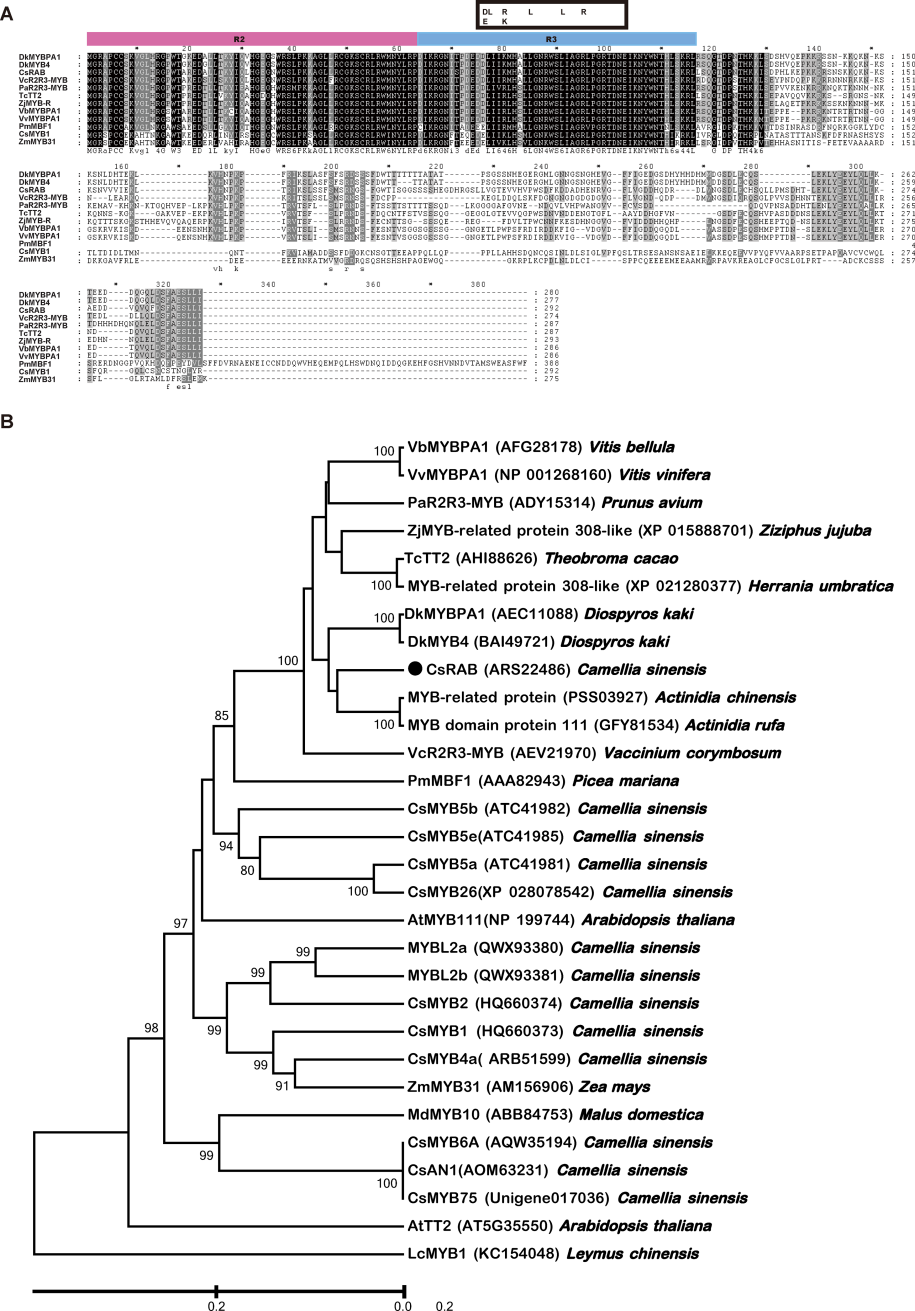
Motif and phylogenetic analysis of *Camellia sinensis* RAB (CsRAB). **(A)** Motif analysis of CsRAB with other MYB transcription factors. The R2 and R3 domains are shown, and the [D/E]Lx2[R/K]x3Lx6Lx3R motif that as predicted to interact with R-like bHLH is indicated by the box. **(B)** Phylogenetic analysis showing the similarities of CsRAB with other MYB transcription factors. The tree was constructed using the linearized neighbor-joining method of MEGA software version 7.0. The scale bar represents 0.2 substitutions per site. The GenBank accession numbers of the MYB transcription factors are as follows: AtMYB111 (NP_199744), AtTT2 (AT5G35550), CsAN1 (AOM63231), CsMYB1 (HQ660373), CsMYB2 (HQ660374), CsMYB4a (ARB51599), CsMYB5a (ATC41981), CsMYB5e (ATC41985), CsMYB6A (AQW35194), CsMYB75(Unigene017036), CsMYB26 (XP_028078542), CsMYB90 (XP 028068990), MYBL2a (QWX93380), MYBL2b (QWX93381), CsRAB (ARS22486), DkMYBPA1 (AEC11088), DkMYB4 (BAI49721), LcMYB1 (KC154048), MdMYB10 (ABB84753), MYB-domain protein 111 (GFY81534), MYB-related protein (PSS03927), MYB-related protein 308-like (XP 021280377), PaR2R3-MYB (ADY15314), PmMYB1 (AAA82943), TcTT2 (AHI88626.1), VbMYBPA1 (AFG28178), VcR2R3-MYB (AEV21970), VvMYBPA1 (NP_001268160), ZjMYB-related protein 308-like (XP_015888701), ZmMYB31 (AM156906).

An RNA-seq analysis was conducted to compare the transcriptomes of the purple and green leaves of variety ‘*Hongfei*’, and an expressed sequence tag (EST) of a *MYB* gene (we named it *CsRAB*)exhibiting a dramatic increase in the purple leaves was identified (https://www.ncbi.nlm.nih.gov/sra/PRJNA828330, PRJNA830308). To confirm the reliability of transcriptomes, we performed qRT-PCR analysis on leaf tissues from variety ‘*Hongfei*’ to examine the transcript abundance of *CsRAB* and five key anthocyanin structural genes encoding *CsANS*, *CsC4H*, *CsCHS*, *CsDFR* and *CsF3H* (**Figure 3A**). The expression levels of those genes were remarkably higher in purple leaves compared with green leaves in ‘*Hongfei*’. Furthermore, the expression of those six genes were also significantly higher in purple leaves of purple variety ‘*Hongfei*’ compared with normal green variety ‘*Yinghong 9*’ (**Figure 3B**). Given the higher anthocyanin accumulations in purple leaves, these data are consistent with the presumption that *CsRAB* plays a positive role in regulating anthocyanin biosynthesis. With the rapid amplification of cDNA ends (RACE) method using the *CsRAB* EST sequence, we obtained the *CsRAB* full-length cDNA. The *CsRAB* cDNA is 1,280 bp, containing an open reading frame (ORF) of 879 bp that encodes a protein of 293 amino acid residues having a molecular mass of 33.14 kDa and a predicted isoelectric point of 9.70. The sequence has been deposited in GenBank (Accession number: KX549467). Database searches revealed that CsRAB shares a high homology with other plant MYB proteins, especially those MYB transcription factors involved in flavonoid synthesis and metabolism. For instance, CsRAB shared an amino sequence identity of 68% with DkMYBPA1 from *Diospyros kaki* and 64% with VcR2R3-MYB from *Vaccinium corymbosum*, respectively. A multiple sequences alignment suggested that CsRAB contains N-terminal R2 and R3 MYB domains that are homologous to the DNA-binding domains in R2R3-MYB proteins of other plants (**Figure 4A**), indicating that CsRAB is a typical R2R3-MYB protein. Additionally, an R-Like bHLH protein-binding site, the [D/E]Lx_2_ [R/K]x_3_Lx_6_Lx_3_R motif, was found in the R3 domain of CsRAB (**Figure 4A**), suggesting that CsRAB may interact with a particular R-Like bHLH protein involved in regulating plant metabolism. Our phylogenetic analysis showed that CsRAB is distantly related to other previously reported MYB proteins involved in anthocyanin biosynthesis and accumulation from tea plants, including CsAN1, CsMYB1, CsMYB2, CsMYB4a, CsMYB5a, CsMYB5e, CsMYB6A, CsMYB75, CsMYB90, CsMYB26, MYBL2a and MYBL2b (**Figure 4B**), which suggests that *CsRAB* is a novel *MYB* gene family member distinct from the reported *C. sinensis MYB* genes participating in regulation of anthocyanins metabolism. The *CsRAB* genomic sequence (2,144 bp) was isolated using a pair of specific primers derived from the start and stop codon regions of the cDNA. Exons 1 (135 bp) and 2 (744 bp) are separated by an intron (1,265 bp) (**Supplementary Figure S1**). The R2 domain-coding region is divided by an intron and spans Exons 1 and 2, whereas the R3 domain-coding region is located within Exon 2 (**Supplementary Figure S1**).

### Sub-cellular localization of the CsRAB protein

**Figure 5:**
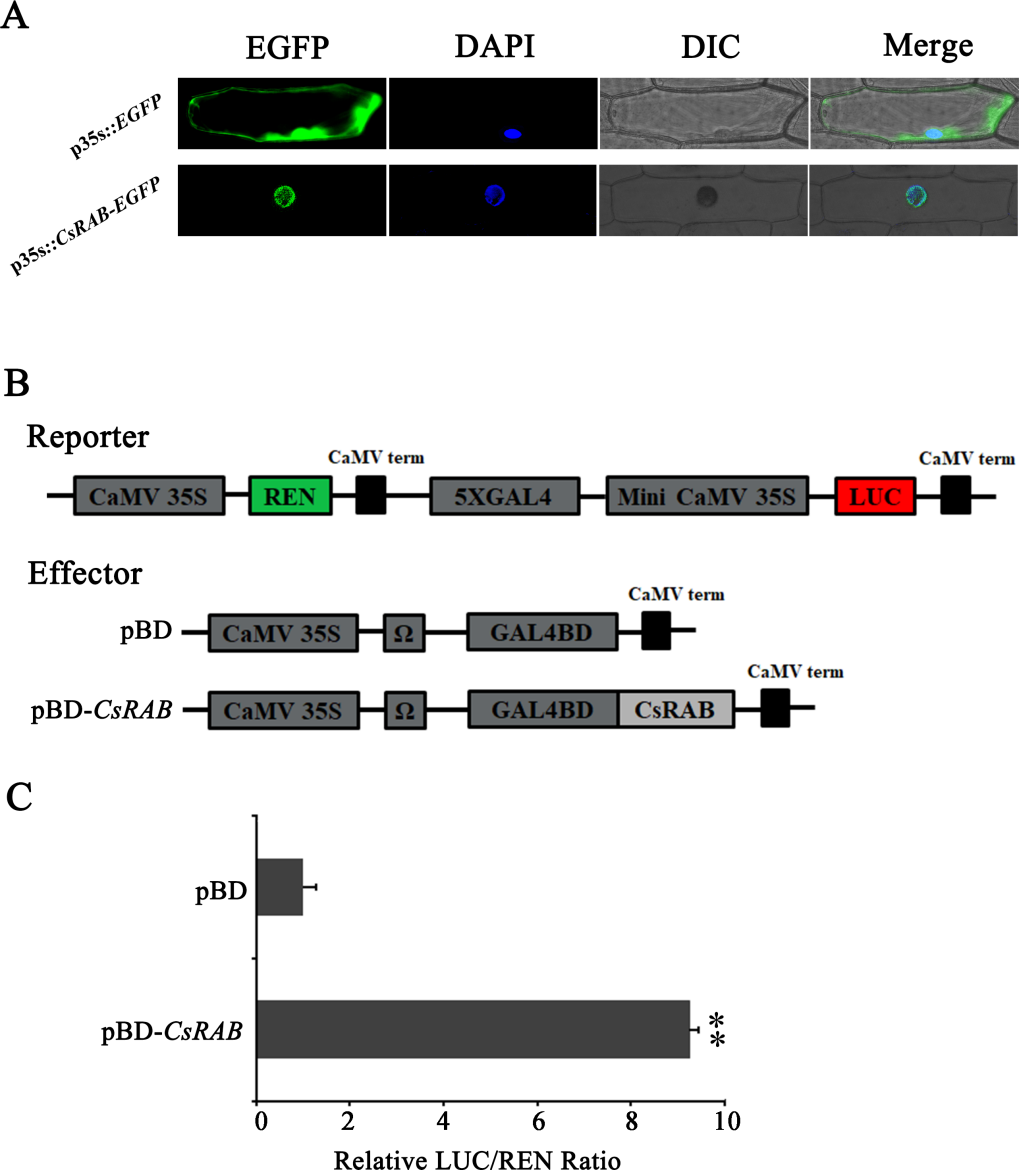
Subcellular localization and transcriptional activation capability of CsRAB. **(A)** Subcellular localization of the CsRAB protein in onion (*Allium cepa*) epidermal cells. For each panel, EGFP: the enhanced green fluorescent image, DAPI: the 6-Diamidino-2’-phenylindole stained image, DIC: the bright field image, Merge: overlaid green fluorescence, DAPI stained and bright-field three images. The images show the nuclear localization of CsRAB. **(B)** The dual renilla luciferase (REN)/luciferase (LUC) reporter and effector constructs. The double-reporter plasmid contained 5× GAL4 and mini-CaMV35S fused to *LUC* and *REN* driven by CaMV35S. The effector plasmid contained the *CsRAB* gene fused to *GAL4BD* driven by CaMV35S. **(C)** The transcription activation ability of CsRAB. The LUC and REN luciferase activities were assayed after the dual REN/LUC reporter and effectors co-transformed into *Arabidopsis* protoplasts 24 h, and the transcription activation capability of CsRAB is indicated by the LUC to REN ratio. Each value represents the means of three biological replicates, and the asterisk indicates a statistically significant difference as assessed by Student’s t-test (^∗∗^P < 0.01) compared with pBD.

To examine the sub-cellular localization of the CsRAB protein, we constructed the vector p35S*::CsRAB*-*enhanced* ***g*** *reen fluorescent protein* (*EGFP*) and transformed it into fresh and healthy onion (*Allium cepa*) epidermal cells, and the pBEGFP expression vector as control. Fluorescence was observed in the nuclei of the p35S*::CsRAB*-*EGFP* transgenic cells, whereas in the control cells, bright green fluorescence was seen throughout (**Figure 5A**), indicating that CsRAB is indeed a nuclear protein that may act as a transcription factor.

### Transcriptional activation activity of CsRAB

The pBD-*CsRAB* construct was generated as an effector (**Figure 5B**). Then the dual renilla luciferase (REN)/luciferase (LUC) reporter and effector constructs were co-expressed in protoplast of *Arabidopsis* by PEG-mediated transformation. As shown in **Figure 4C**, compare to the vector-only pBD control, pBD-*CsRAB* significantly activated the expression of the LUC reporter, and the LUC/REN ratio of pBD-*CsRAB* was 9.2-fold higher than that of the control (pBD) (**Figure 5C**). These results suggest that CsRAB has transcriptional activation activity *in vivo*, and may act as a transcriptional activator.

### Overexpression of *CsRAB* in transgenic *Arabidopsis thaliana*

To the best of our knowledge, no effective transformation system has been established in tea plants. Therefore, to confirm the function of *CsRAB* in anthocyanin biosynthesis, the *CsRAB* ORF (driven by a constitutive promoter) was introduced into the model plant *Arabidopsis* by *Agrobacterium tumefaciens-*mediated transformation. Several independent transgenic *Arabidopsis* lines were obtained.

**Figure 6:**
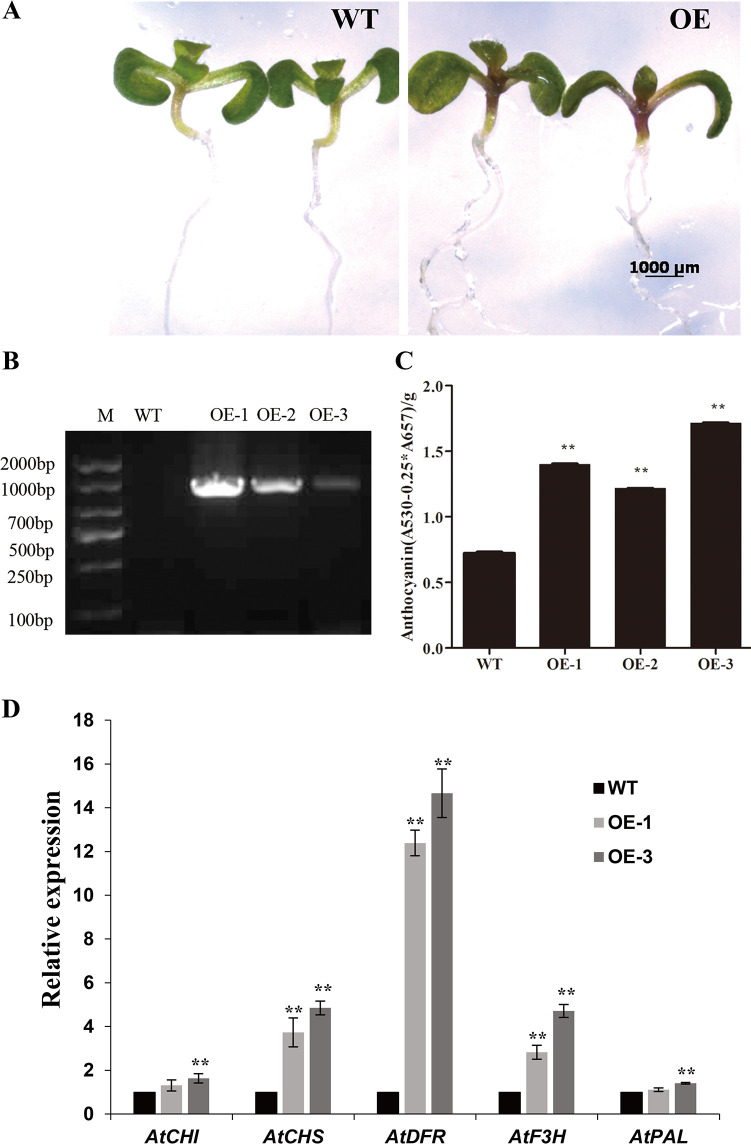
Phenotype and expression analysis of the structural genes in the anthocyanin biosynthetic pathway of transgenic *Arabidopsis* seedlings. **(A)** Comparison of wild type (WT) and transgenic *Arabidopsis* seedlings at 6-day after germination. Note the purple transgenic stems; **(B)** Detection of *CsRAB* transcript in Col-0 WT and *CsRAB*-overexpressing lines OE-1, OE-2 and OE-3 by PCR; **(C)** Soluble anthocyanin contents in the *CsRAB*-overexpressing lines and WT. **(D)** The expression levels of structural genes in the anthocyanin biosynthetic pathway in WT and *CsRAB*-overexpressing seedlings at 6 d after germination. Except for *AtPAL* and *AtCHI*, expression levels of other the structural genes were higher in the *CsRAB*-overexpressing lines than in the WT. Data presented are mean values of three biological repetitions. The asterisk indicates a statistically significant difference as assessed by Student’s t-test (^∗∗^P < 0.01) compared with WT.

To examine the potential differences between the transgenic and wild type (WT) plants, seeds from three randomly selected transgenic lines (OE-1, OE-2 and OE-3) and WT were sown simultaneously on MS medium without antibiotics in one Petri dish. Morphologically, there were no obvious differences between the transgenic lines and WT. However, as shown in **Figure 6A**, dark purple stems were observed in 6-d-old transgenic seedlings, while the WT seedlings were still green (**Figure 6A**, **Supplementary Figure S2**). The transgenic plants were verified through PCR amplification using *CsRAB* gene specific primers TF and TR (**Supplementary Table S1**). *CsRAB* expression was observed in the three transgenic lines examined but not in the WT (**Figure 6B**). To investigate whether the stem color change was a result of elevated anthocyanin levels, the anthocyanin contents in stems were analyzed (**Figure 6C**). The anthocyanin contents in all the selected transgenic lines were approximately two folds higher than in the WT control, indicating that *CsRAB* expression in *Arabidopsis* seedlings promoted anthocyanin accumulation.

**Figure 7:**
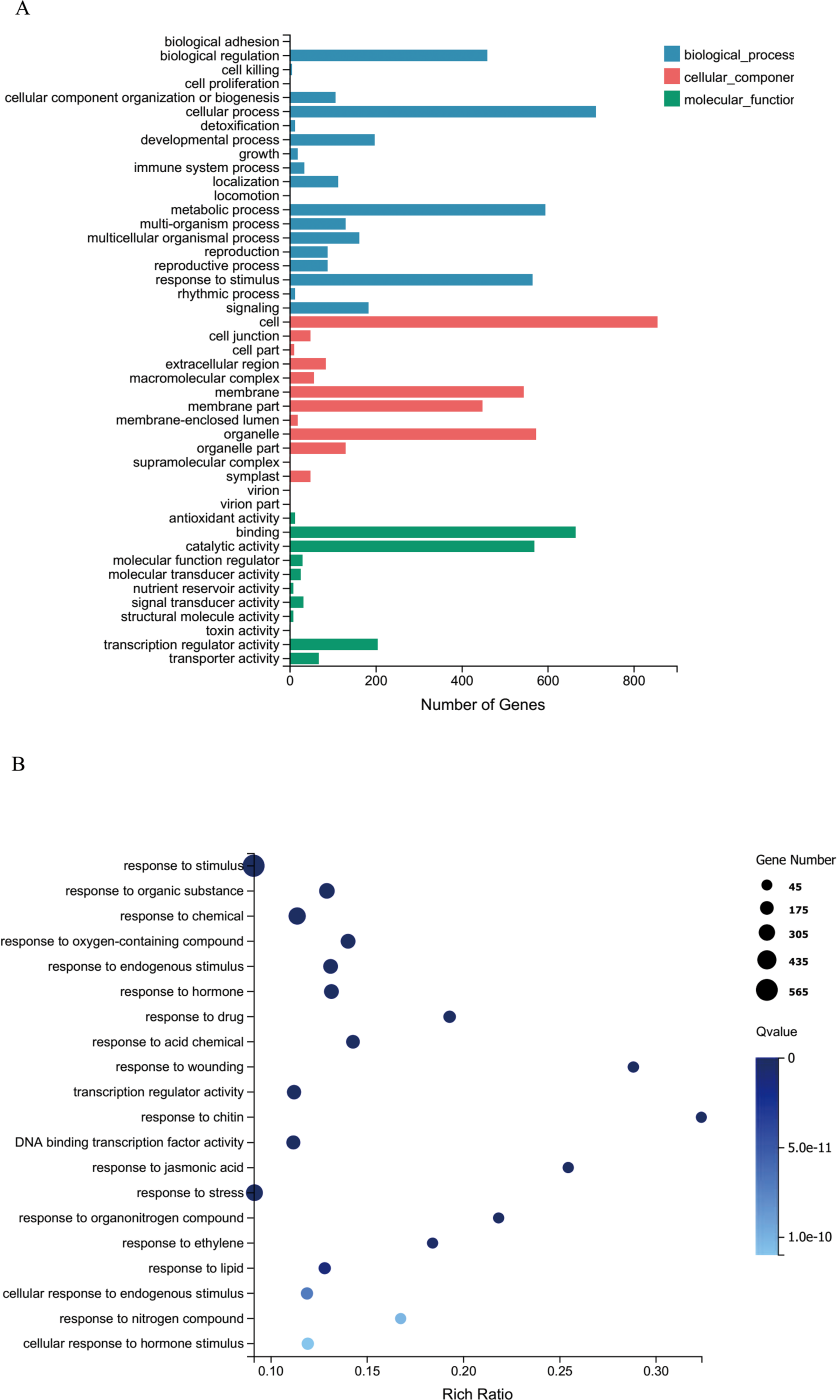
Gene ontology (GO) enrichment analysis of differentially expressed genes (DEGs) in WT and *CsRAB*-overexpressing *Arabidopsis* transcriptome. **(A)** Classification of GO-enriched DEGs. The genes were assigned to three main categories, biological process, cellular component and molecular function. The numbers of DEGs and the names of the GO categories are presented along the x- and y-axes, respectively. **(B)** Scatterplot of GO-enriched functions of DEGs. The enrichment factor is the ratio of the DEG number to the total gene number in a certain pathway. The colors and sizes of the dots represent the q-value range and gene number, respectively.

**Figure 8:**
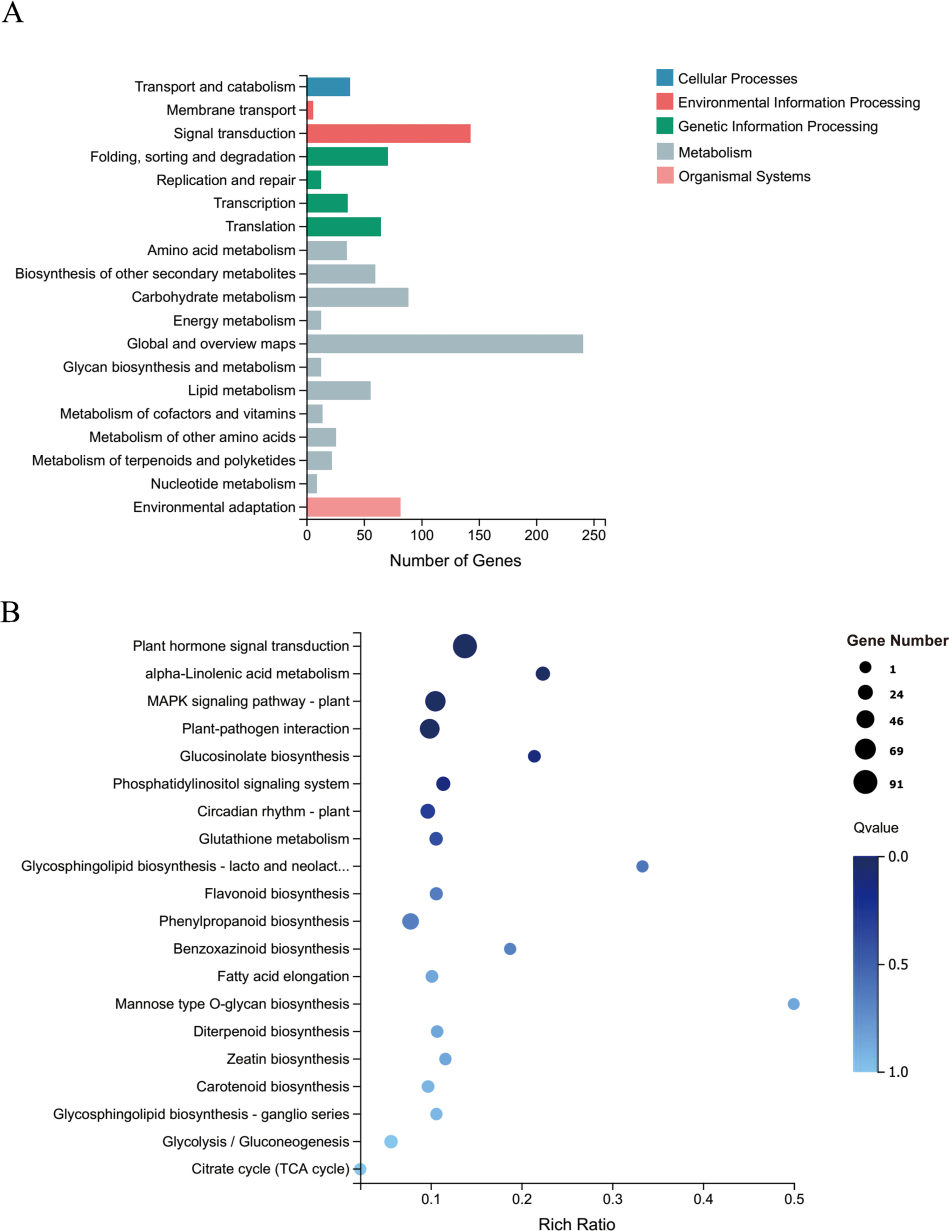
Kyoto encyclopedia of genes and genomes (KEGG) pathway enrichment analysis of DEGs in WT and *CsRAB*-overexpressing *Arabidopsis* transcriptome. **(A)** Classification of KEGG pathways enriched with DEGs. The numbers of DEGs and the names of the KEGG categories are presented along the x- and y-axes, respectively. **(B)** Scatterplot of KEGG pathways enriched for DEGs. The enrichment factor is the ratio of the DEG number to the total gene number in a certain pathway. The colors and sizes of the dots represent the q-value range and gene number, respectively.

To further explore the functions of *CsRAB* in *Arabidopsis*, we examined transcriptome changes in *CsRAB*-overexpressing *Arabidopsis* plants through an RNA-seq analysis. Compared with WT plants, the expression levels of 1,552 genes were altered, with 965 genes being up-regulated, and 587 genes being down regulated in *CsRAB*-overexpressing plants (**Supplementary Table S2**). This suggested that *CsRAB* acts mainly as a transcriptional activator. The major functions of the differentially expressed genes were analyzed using a Gene Ontology (GO)-enrichment analysis, as shown in **Figures 7A, B**. The GO-enrichment analysis revealed that the genes modulated by *CsRAB* are involved in multiple aspects of biological processes, including ‘cellular process’, ‘metabolic process’ and ‘response to stimulus’, also including ‘cell, organelle, and membrane’, and further including ‘binding, catalytic activity’ and ‘transcription regulator activity’ in the three categories, biological process, cellular component, and molecular function, respectively (**Figures 7A, B**). In addition, a Kyoto Encyclopedia of Genes and Genomes (KEGG) pathway enrichment analysis showed that these differentially expressed genes were mainly involved in ‘Plant hormone signal transduction’, ‘alpha-Linolenic acid metabolism’, ‘mitogen-activated protein kinase signaling pathway’, ‘Plant-pathogen interaction’, ‘Glucosinolate biosynthesis’, ‘Phosphatidylinositol signaling system’ and ‘Circadian rhythm’ (**Figures 8A, B**).

Seven structural genes, including *PAL, CHS, CHI, F3H, F3’H, DFR*, and *ANS*, which act in the anthocyanin biosynthetic pathway, have been well characterized in plants previously (Holton and Cornish, 1995). Here, the RNA-seq data revealed that a number of structural genes involved in anthocyanin biosynthesis such as *AtCHS*, *AtDFR*, *AtF3H* were up-regulated in *CsRAB*-overexpressing plants. To confirm the reliability of differentially expressed genes, two transgenic *CsRAB*-overexpressing lines with higher anthocyanin accumulation were selected. We further performed qRT-PCR to analyze the expression levels of anthocyanin biosynthetic genes. As shown in **Figure 6D**, the expression levels of *AtCHS* and *AtDFR* were significantly higher than in WT seedlings, which was consistent with the RNA-seq data. Furthermore, the qRT-PCR analysis also demonstrated that the transcript levels of *AtF3H* were higher in *CsRAB*-overexpressing plants. Additionally, slightly higher expression of *AtPAL* and *AtCHI* only detected in one overexpressing line, so there were no significant differences in *AtPAL* and *AtCHI* expression levels between *CsRAB-*overexpressing and WT seedlings (**Figure 6D**). Thus, these results indicating that *CsRAB* may play important roles in anthocyanin biosynthesis, as well as in multiple aspects of plant development and plant response to stimuli.

## Discussion

In recent years, there has been growing interest in cultivating purple tea containing high anthocyanin contents, because of their positive effects on human health. However, the transcriptional regulatory mechanisms underlying the anthocyanin accumulation in purple tea are currently unclear. Anthocyanins are pigments that give plants colors ranging from red to purple. The dominant anthocyanin, Cyanidin-*O*-syringic acid, in ‘*Hongfei*’ maybe contribute to its distinct red purple hue, which is different from ‘*Zijuan*’ with dark purple tender shoots. On the basis of this study, we isolated a R2R3-MYB gene from ‘*Hongfei*’ with red purple color, *CsRAB*, which harbors an ORF of 879 bp encoding 293 amino acid residues. An analysis of the deduced amino acid sequence revealed that CsRAB contains an N-terminal R2R3 repeat that is homologous to the DNA-binding domains of other plant R2R3-MYB proteins, whereas the C-terminal region has little homology to other MYBs (**Figure 4A**). A comparison of the CsRAB protein sequence with some MYB proteins from other plants deposited in Genbank indicated that its sequence is significantly similar to DkMYB4 and DkMYBPA1 (**Figure 4B**). In *Arabidopsis* and rice, the R2R3-MYB subfamily, which mainly participates in regulating flavonoid metabolism, contains the largest numbers of *MYB* genes (Chen et al., 2006). However, the expression patterns and functional properties of MYBs vary a lot among different plants and even in different tissues of the same plant. In tea plants, correlations between the expression levels of some R2R3-MYB transcription factors and anthocyanin contents have been reported (He et al., 2018; Jiang et al., 2018; Li et al., 2017; Ma et al., 2012; Sun et al., 2016; Wang et al., 2018; Wei et al., 2019; Zheng et al., 2019).

Although gene sequence homology and expression pattern analysis provide important clues for predicting gene functions, only functional analysis is effective in revealing the roles of genes in plant development. However, there is no effective transformation system currently available for tea plants; therefore, the model plant *Arabidopsis* was used as a heterologous host to further analyze the functions of flavonoid metabolism-related genes. The nuclear subcellular localization and transcriptional regulatory activity assay indicated that CsRAB has transcriptional activation activity *in vivo*. To further analyze the functions of *CsRAB*, we examined the consequences when it was overexpressed in *Arabidopsis*. We observed purple stems in *CsRAB*-overexpressing plants, which had significantly higher anthocyanin contents than WT stems. This study identified and characterized a novel *MYB* gene distantly related to other previously reported MYB proteins involved in regulating anthocyanin biosynthesis in tea plants, and the results contribute to our understanding of the molecular mechanisms controlling secondary metabolism and the development of purple buds and leaves in tea plants.

The first plant *MYB* gene involved in anthocyanin synthesis, *C1*, was identified in *Zea mays by* Paz-Ares et al. in 1987. Since then, a large of number of MYB transcription factors regulating anthocyanin metabolism have been found in many plants. For instance, three R2R3-MYB transcription factors MYB11, MYB12, and MYB111 regulate the biosynthesis of flavonol through the activation of CHS, CHI, and F3H, whereas PAP1**/**MYB75, PAP2/MYB90, MYB113 and MYB114 identified as R2R3-MYB transcription factors to compose MYB-bHLH-WD40 (MBW) complex to regulate anthocyanin-specific biosynthetic pathway in *Arabidopsis thaliana* (Borevitz et al., 2000; Gonzalez et al., 2008). In grape berry (*V. vinifera* L.), both *VvMYBA1* and *VvMYBA2* can regulate colour synthesis in the transient assay of grape cells; furthermore, *VvMYBA* genes led to the induction *UDP-glucose: flavonoid 3-O-glucosyltransferase* gene expression and the accumulation of anthocyanin (Kobayashi et al., 2002; Walker et al., 2010). Additionally, transcription factor CsMYB75 and glutathione transferase CsGSTF1 expression are correlated with anthocyanin accumulation in tea plants. CsMYB75 promotes the expression of *CsGSTF1* in tobacco, and *CsGSTF1* restores anthocyanin accumulation in an *Arabidopsis* mutant (Wei et al., 2019). Most of the MYBs act as transcriptional activators in biosynthesis of flavonoids including anthocyanins; however, a few MYB members function as transcriptional repressors in flavonoid synthesis. For example, a R2R3-MYB transcription factor from fruit of commercial strawberry (*Fragaria ananassa*), FaMYB1, acts as a transcriptional repressor, and its heterologous over-expression in tobacco down-regulates the expression of some structural genes, resulting in decreased anthocyanin accumulation (Aharoni et al., 2001). In the present study, *CsRAB*, encoding a R2R3-MYB transcription factor, was heterologously overexpressed in *Arabidopsis*, which led to significant anthocyanin accumulation in the stems. In addition, the dual-luciferase reporter assay revealed that CsRAB has transcriptional activation activity *in vivo*. Collectively, these results indicated that *CsRAB* functions positively in regulating anthocyanin synthesis.

In the dicot anthocyanin biosynthetic pathway, the early biosynthesis genes are activated by independent R2R3-MYB transcription factors, whereas late biosynthesis genes require a co-activator, such as the MBW transcription factors complex (Petroni and Tonelli, 2011). Here, the expression levels of both early and late biosynthesis genes including *AtCHS*, *AtF3H* and *AtDFR* in the anthocyanin synthesis pathway increased in *CsRAB*-overexpressing transgenic *Arabidopsis* seedlings, indicating that *CsRAB* specifically elevated the anthocyanin contents by promoting the transcription of these genes. Furthermore, the bHLH protein-interaction motif [D/E]Lx_2_ [R/K]x_3_Lx_6_Lx_3_R was found in the R3 domain of CsRAB (**Figure 4A**), and it may potentially contribute to the specificity of the interaction between CsRAB and its bHLH copartner. However, more in-depth studies are needed to determine whether the formation of MBW complex between CsRAB, bHLH and WD40 is required for regulating anthocyanin biosynthesis genes. In addition, the expression levels of *AtPAL* and *AtCHI*, which encode the enzymes that catalyze the early steps in the biosynthesis of phenylpropanoids including lignins, flavonoids, coumarins, and stilbenoids, were unchanged between the transgenic lines and WT. Therefore, it is likely that *AtPAL* and *AtCHI* are not regulated by *CsRAB* in *Arabidopsi*s.

In summary, we successfully identified a new R2R3-MYB transcription factor gene, *CsRAB*, from the purple tea. On the basis of the *CsRAB*-overexpressing *Arabidopsis* plants’ phenotype, as well as the specific up-regulation of the anthocyanin synthesis genes in the transgenic plants, we propose that CsRAB acts as a transcription factor to positively regulate anthocyanin biosynthesis. Thus, the results have broadened our understanding of the molecular regulation of anthocyanin biosynthesis in *C. sinensis*.

## Data Availability

The datasets presented in this study can be found in online repositories. The names of the repository/repositories and accession number(s) can be found below: https://www.ncbi.nlm.nih.gov/, PRJNA828330 https://www.ncbi.nlm.nih.gov/, PRJNA830308.
