# Supplementary material for: CsRAB, a R2R3-MYB transcription factor from purple tea (Camellia sinensis), positively regulates anthocyanin biosynthesis
Source: Front Plant Sci. 2024 Dec 6;15:1514631. doi: 10.3389/fpls.2024.1514631 (PMC11658990; doi:10.3389/fpls.2024.1514631)
Supplement: Supplementary file 1 [file DataSheet1.pdf]

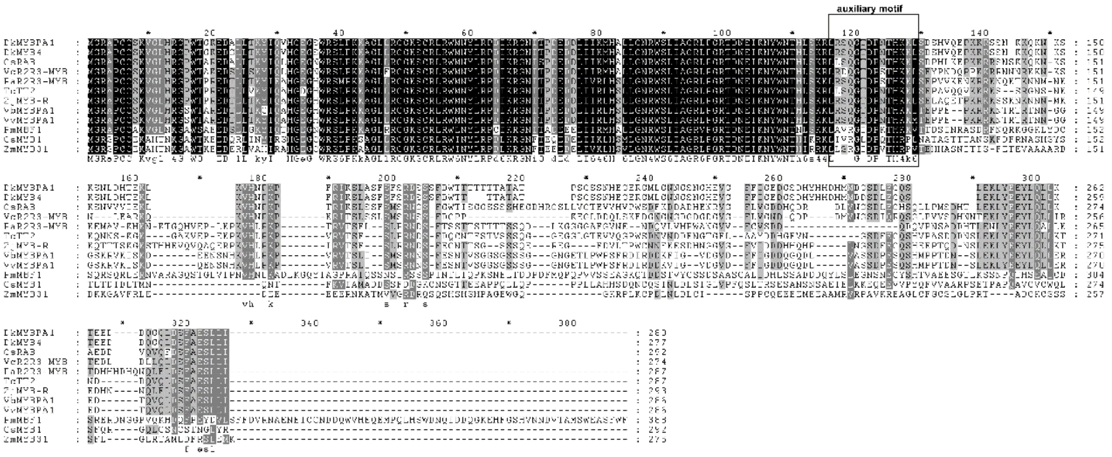

Figure S1. Motif analysis of full length of the CsRAB protein. Auxiliary motif of R2R3-MYB is indicated by box.

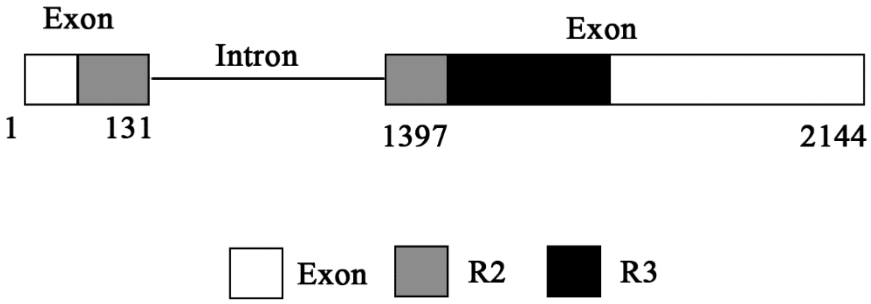

Figure S2. Structure of the *CsRAB* gene. Exons and introns are labeled. Numbers refer to position relative to the first nucleotide of the start codon. Exons are indicated by boxes, and introns are indicated by lines. Locations of R2 and R3 domains are highlighted with gray and black backgrounds, respectively.

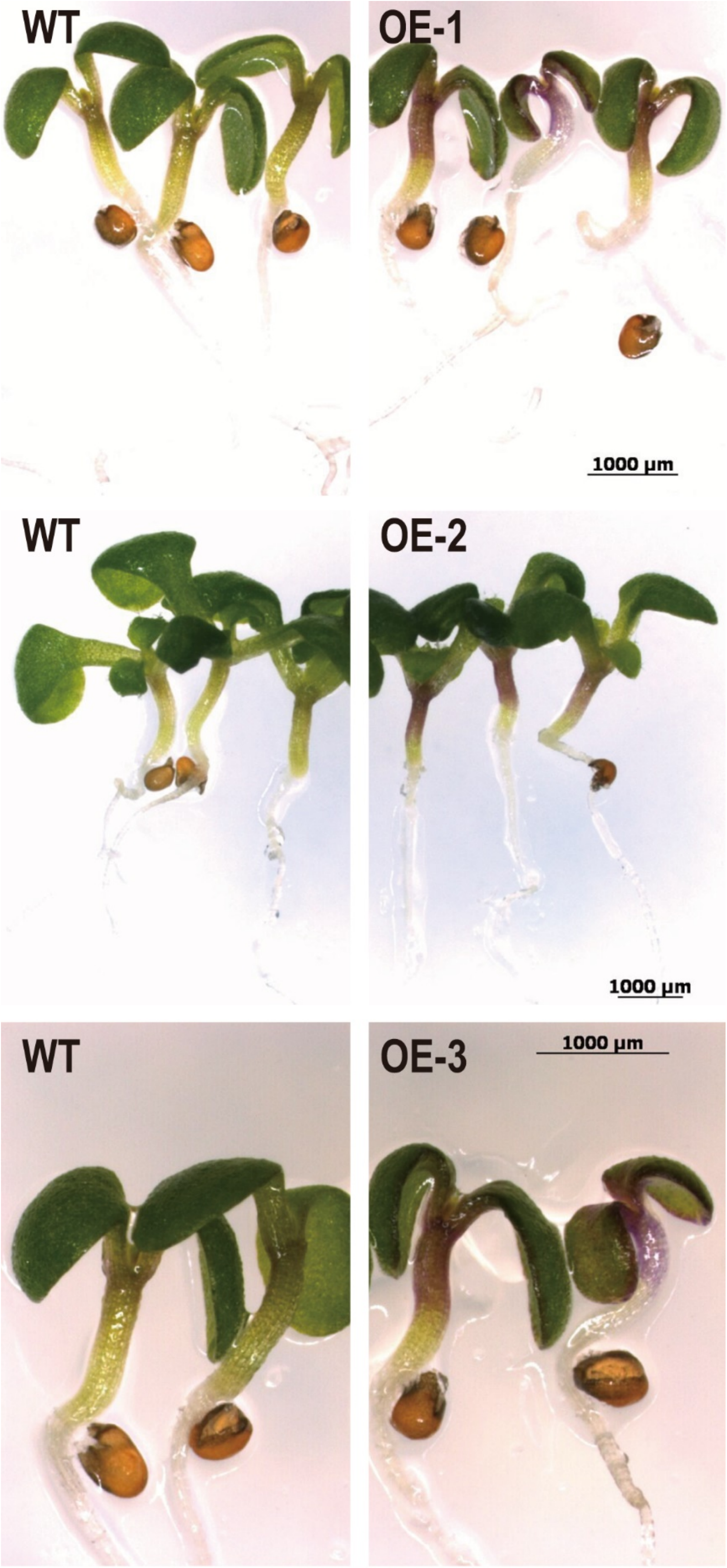

Figure S3. Phenotypic comparison of wild type (WT) and *CsRAB*-overexpressing lines OE-1, OE-2 and OE-3 at 6-day after germination.
